# Case Report: *Vibrio fluvialis* isolated from a wound infection after a piercing trauma in the Baltic Sea

**DOI:** 10.1099/acmi.0.000312

**Published:** 2022-01-12

**Authors:** Juliane Hecht, Maria Borowiak, Bernhard Fortmeier, Salah Dikou, Wolfgang Gierer, Ingo Klempien, Jonas Nekat, Stephan Schaefer, Eckhard Strauch

**Affiliations:** ^1^​ Unfallchirurgie, Helios Hanseklinikum Stralsund, Große Parower Straße 47-53 18435, Stralsund, Germany; ^2^​ German Federal Institute for Risk Assessment, BfR, Max-Dohrn-Str. 8-10, 10589 Berlin, Germany; ^3^​ MVZ Limbach Vorpommern Rügen, Große Parower Str. 47-53, 18435 Stralsund, Germany; ^4^​ Klinische Hygiene und Infektiologie, Helios Hanseklinikum Stralsund, Große Parower Straße 47-53 18435 Stralsund, Germany

**Keywords:** *Vibrio fluvialis*, wound infection, Baltic Sea, complete genome sequence

## Abstract

*
Vibrio* spp. are Gram-negative bacteria found in marine ecosystems. Non-cholera *Vibrio* spp. can cause gastrointestinal infections and can also lead to wound infections through exposure to contaminated seawater. *Vibrio* infections are increasingly documented from the Baltic Sea due to extended warm weather periods. We describe the first isolation of *Vibrio fluvialis* from a wound infection acquired by an impalement injury in the shallow waters of the Baltic Sea. The severe infection required amputation of the third toe. Whole genome sequencing of the isolate was performed and revealed a genome consisting of two circular chromosomes with a size of 1.57 and 3.24 Mb.

## Introduction

*Vibrio (V*.) spp. are halophilic rod-shaped bacteria found in marine waters [1]. Of the approximately 100 known *Vibrio* species at least twelve have been linked to human infections [1]. Strains belonging to the serogroups non-O1/non-O139 of *V. cholerae* are the causative agent of cholera, an epidemic diarrheal disease which still is a great public health problem in many developing countries. Other human pathogenic *Vibrio* isolates (designated as non-cholera *Vibrio* spp.) mainly can cause gastrointestinal infections and wound infection infections. *V. vulnificus* in particlar is a feared pathogen causing severe wound infections and bacteremia with high mortality [2]. Other non-cholera *Vibrio* spp. seem to be less virulent than *V. vulnificus *. Wound infections have been described after infection with *V. parahaemolyticus, V. alginolyticus* [1] and *V. harveyi* [3].

Although located at high latitude, the Baltic Sea offers long stretches of shallow coastal regions, where water temperature in summer allows growth of *Vibrio* spp. Already in 1992 *V *. *anguillarum* has been isolated from the Baltic Sea [4]. Later on, *V. alginolyticus, V. cholerae* (non-O1 and non-O139), *V. mimicus, V. parahaemolyticus* and *V. vulnificus* have been found in brackish waters of the Baltic Sea [5–7] and *V. diazotrophicus* in deep subsurface sediments [8].

Vibriosis, severe wound infections and septicemias after contact with the Baltic Sea have been described mainly for *V. vulnificus* e.g. [9]. Less severe infections with *V. parahaemolyticus* and non-O1/non-O139 *V. cholerae* are increasingly being observed around the Baltic Sea [10, 11].

*V. fluvialis* has been recognized as an emergent pathogen mainly causing diarrhoea. Single reports of bacteremia, endophthalmitis, cholangitis and cerebritis have been reviewed [12]. *V. fluvialis* has been retrieved from marine sources in Asia, America, Africa and from the Mediterranean Sea [12] but to our knowledge rarely from the Baltic Sea. We describe the first isolation of *V. fluvialis* from a wound infection acquired by an impalement injury caused by penetration of an extremity (foot) with a piercing object.

## Case report, treatment and outcome

On 4 August 2019, a male patient (49 years old) dismounted his surfboard in the shallow waters of the strait between Stralsund and the island of Rügen. He stepped onto an unknown piercing object and suffered an impalement injury of his left foot. The water temperature in Kramerhof (close to Stralsund) had been reported as 21 °C on August 4^th^ after a sunny period of warm days with temperature highs of around 25 °C.

In our hospital the heavily contaminated wound between the second and third metatarsal bones was cleaned. After debridement a wick was inserted. Standard antibiotic therapy was initiated with ampicillin/sulbactam 3 g three times a day, i.v. Because of presumed uncomplicated wound conditions, no swabs for microbiological investigations were taken.

Due to an increasing infection of the soft tissue the wound was revised on day 6. After debridement negative pressure wound therapy was applied. A swab taken from the phlegmonous tissue grew *V. fluvialis* on day 8. The initial antibiotic susceptibility testing showed sensitivity to ampicillin/sulbactam, thus therapy was continued. When changing the negative pressure wound therapy on day 9, a drastic worsening was noticed. Intensive debridement had to be performed and thereafter polihexanid lavasorb bandage was applied. Due to the worsened infection antibiotic therapy was switched to ceftriaxone and ciprofloxacin to combat the bacterial infection. The biopsies taken during surgery grew *Enterobacter cloacae* and *Proteus vulgaris* on day 10 of which only *P. vulgaris* was intermediary susceptible to ampicillin/sulbactam acid. Both isolates were sensitive to ciprofloxacin. On day 10 the amputation of the third toe became necessary due to massive bone and soft tissue necrosis. After amputation the clinical situation improved, and the patient left the hospital on day 17. Ceftriaxone i.v. was discontinued on day 12, oral ciproxfloxacin therapy ended on day 18.

### Microbiological diagnosis

Colonies derived from the phlegmonous soft tissue during the first wound revision were grown on sheep blood agar and identified by matrix-assisted-laser-desorption/ionization time-of-flight mass spectrometry (MALDI-TOF MS) as *V. fluvialis *. Antibiotic susceptibility testing (EUCAST) had been done in Vitek 2 (Biomérieux) at first and revealed an isolate widely sensitive to ampicillin, ceftriaxone, ceftazidime, ciprofloxacin, trimethoprim/sulfamethoxazole and doxycycline.

However, the antibiotic susceptibility testing of the *V. fluvialis* isolate was repeated later with the standard Kirby–Bauer disc diffusion method following Clinical and Laboratory Standards Institute (CLSI) guidelines. The isolate was found to be resistant to 10 μg per disc of ampicillin, and sensitive (μg per disc) to ceftriaxone (30), ciprofloxacin (5), chloramphenicol (30), trimethoprim/sulfamethoxazole (1.25/23.75), tetracycline (30) and gentamicin (30).The isolate was sent to the *Vibrio* reference laboratory at the German Federal Institute for Risk Assessment for species confirmation and was confirmed as *V. fluvialis* by using MALDI-TOF MS. A number of phenotypical tests were carried out und showed the expected results for the species (according to ISO standard 21872-2 : 2006). The isolate 19-VB00936 was oxidase-positive and required NaCl for growth in 1% peptone water (PW). Colonies on thiosulfate-citrate-bile salts (TCBS) agar were yellow and no production of gas from glucose fermentation was observed (discrimination to the species *V. furnissii *). Lysine-decarboxylase and ornithine-decarboxylase tests were negative and arginine-dihydrolase test positive. Fermentation of sucrose was positive and for lactose negative, ONPG hydrolysis was positive.

### Molecular characterization

A conventional *toxR* PCR for species determination of *V. fluvialis* was performed [13] and yielded a PCR product with the expected size. Finally, sequence analysis of an 871 bp fragment of the coding region of the *rpoB* gene was carried out which is a valuable tool for species identification [14–16]. The obtained sequence revealed identity to several *V. fluvialis rpoB* sequences of more than 99%.

Moreover, the isolate was subjected to whole genome sequencing for further investigation. Therefore, the isolate was cultivated for 24 h at 37 °C in lysogeny broth. Genomic DNA was extracted using the PureLink Genomic DNA Mini Kit (Thermo Fisher Scientific, Waltham, MA, USA) and sequenced using Illumina short-read (Illumina, San Diego, CA, USA) and Oxford Nanopore Technologies (ONT, Oxford, UK) long-read sequencing platforms.

An Illumina sequencing library was prepared using the Nextera DNA Flex Kit. Paired-end sequencing was performed in 2×151 bp cycles on an Illumina iSeq instrument using the iSeq 100 i1 Reagent v1 Kit (300-cycle). An ONT sequencing library was prepared using the Rapid Barcoding Kit and sequenced on an ONT MinION Mk1C sequencer using a Flongle adapter and a Flongle flowcell (FLO-FLG001).

Illumina short-reads were trimmed using fastp v0.19.5 [17]. ONT long-reads were trimmed using Porechop v0.2.3 (https://github.com/rrwick/Porechop), filtered using NanoFilt v2.7.1 and quality checked using NanoStat v1.4.0 [18]. Illumina and ONT reads were combined in a hybrid assembly using Unicycler v0.4.8 including Pilon [19–21]. The assembly resulted in two circular bacterial chromosomes chr_1 (3237604 bp) and chr_2 (1570538). The overall G+C content of the bacterial genome was 50.1%. The genome sequence was deposited in the NCBI nucleotide database. Search for antimicrobial resistance (AMR) genes and point mutations was conducted using AMRFinderPlus v3.10.1 with database version 2021-03-01.1 [22]. No AMR genes or point mutations associated with AMR could be detected. The genome sequence was uploaded to the PATRIC Bioinformatics Resource Centre [23]. The closest reference genome to 19-VB00936 was identified using the Similar Genome Finder Tool searching the reference and representative genomes database. The closest reference identified was *Vibrio fluvialis* strain ATCC 33809 (FDAARGOS_104, NCBI BioSample: SAMN03996321) isolated from a patient from Bangladesh suffering from a *Vibrio* infection. The two genome sequences were compared using the ANI calculator from Kostas lab (http://enve-omics.ce.gatech.edu/ani/index). An Average Nucleotide Identity (ANI) of 98.46% was calculated.

## Discussion

*Vibrio* spp. need water temperatures of around 20 °C to promote growth [7]. Thus, growth of *Vibrio* and infections thereof are frequently encountered in warm equatorial oceans [1]. The Baltic Sea is located at a relatively high latitude with freezing temperatures in some winters. Nevertheless, *Vibrionaceae* have been described as early as 1992 in the Baltic Sea [4]. Even more, due to climate change a warming trend is evident in the Baltic region [24] and an increasing number of reports have shown a rising number of severe infections with *V. vulnificus* in shallow coastal regions of the Baltic Sea in warm summers [9, 10].

We report a soft tissue infection with *V. fluvialis* after an impalement injury acquired in the Baltic Sea. To our knowledge, this is the first reported wound infection with of *V. fluvialis* in the Baltic Sea. *V. fluvialis* infections generally seem to be less severe than infections with *V. vulnificus* [1, 12], however, wound infections have been described in case reports from Taiwan [25] and the USA [26].

If *V. fluvialis* in our case report only caused a soft tissue infection or was paramount to the development of bone infections and necrosis leading to amputation of the third toe remains elusive. *V. fluvialis* was the only bacterium identified after 7 days of antibiotic therapy. However, materials retrieved while amputating the third toe only gave growth to *P. mirabilis* and *E. cloacae *.

What can be learnt from this case report? The standard therapy with ampicillin/sulbactam was suboptimal to cure the heavily contaminated wound. In hindsight, therapy of a deep-seated infection acquired in shallow waters of the Baltic Sea in a particularly hot season would have warranted the consideration of *Vibrio* spp. in the choice of the antibiotic therapy. Most reviews and the CDC suggest third generation cephalosporins for the treatment of *V. vulnificus* infections either as single therapy or in combination with doxycycline or ciprofloxacin [1, 27]. Whether therapy with a third-generation cephalosporin would have prevented the amputation remains unknown.
